# Lifestyle, Demographic and Socio-Economic Determinants of Mental Health Disorders of Employees in the European Countries

**DOI:** 10.3390/ijerph191911913

**Published:** 2022-09-21

**Authors:** Dawid Majcherek, Arkadiusz Michał Kowalski, Małgorzata Stefania Lewandowska

**Affiliations:** 1 Department of International Management, Collegium of World Economy, SGH Warsaw School of Economics, al. Niepodległości 162, 02-554 Warsaw, Poland; 2 World Economy Research Institute, Collegium of World Economy, SGH Warsaw School of Economics, al. Niepodległości 162, 02-554 Warsaw, Poland

**Keywords:** mental disorders, mental health, lifestyle, demographic, socio-economic status, gradient-boosted trees, European countries, European Health Interview Survey

## Abstract

Ensuring the health and well-being of workers should be a top priority for employers and governments. The aim of the article is to evaluate and rank the importance of mental health determinants: lifestyle, demographic factors and socio-economic status. The research study is based on EHIS 2013–2015 data for a sample of N = 140,791 employees from 30 European countries. The results obtained using machine learning techniques such as gradient-boosted trees and SHAPley values show that the mental health of European employees is strongly determined by the BMI, age and social support from close people. The next vital features are alcohol consumption, an unmet need for health care and sports activity, followed by the affordability of medicine or treatment, income and occupation. The wide range of variables clearly indicates that there is an important role for governments to play in order to minimize the risk of mental disorders across various socio-economic groups. It is also a signal for businesses to help boost the mental health of their employees by creating holistic, mentally friendly working conditions, such as offering time-management training, implementing morning briefings, offering quiet areas, making employees feel valued, educating them about depression and burnout symptoms, and promoting a healthy lifestyle.

## 1. Introduction

The ability of an individual to lead a fulfilling life, including the ability to study, work or pursue leisure interests, as well as the ability to make daily personal or household decisions about education, employment, housing or other choices, is heavily influenced by their mental health or psychological well-being [1]. The review of the literature reveals that mental health is a function of two independent but interrelated domains, including positive mental health (PMH) and the presence or absence of mental disorders [2], with the latter being defined as “clinically significant disturbance in an individual’s cognition, emotional regulation, or behaviour” [3]. Mental health and mental disorders are not, therefore, two ends of the same spectrum, and the absence of mental disorder is not the only necessary condition for good mental health [2,4].

Mental health issues have severe financial repercussions for businesses and the economy as a whole. According to survey data from the United Kingdom, Germany and Australia, depression and anxiety are more prevalent in working age than in later life. They account for a large proportion of disability and impose significant economic costs and financial losses on governments around the world [5]. According to the Technical Officer for the International Labour Organisation in Geneva, lost productivity due to mental health issues at work costs the world economy up to 1 trillion USD annually [6].

The COVID-19 epidemic has also resulted in considerable changes in the terms and circumstances of employment for employees, creating new psychosocial challenges that endanger their health and wellbeing. For instance, during the pandemic in Switzerland, the monthly cost of work-related stress dramatically increased by 600 million CHF each month from 7.6 billion CHF before pandemic time [6]. 

**Figure 1:**
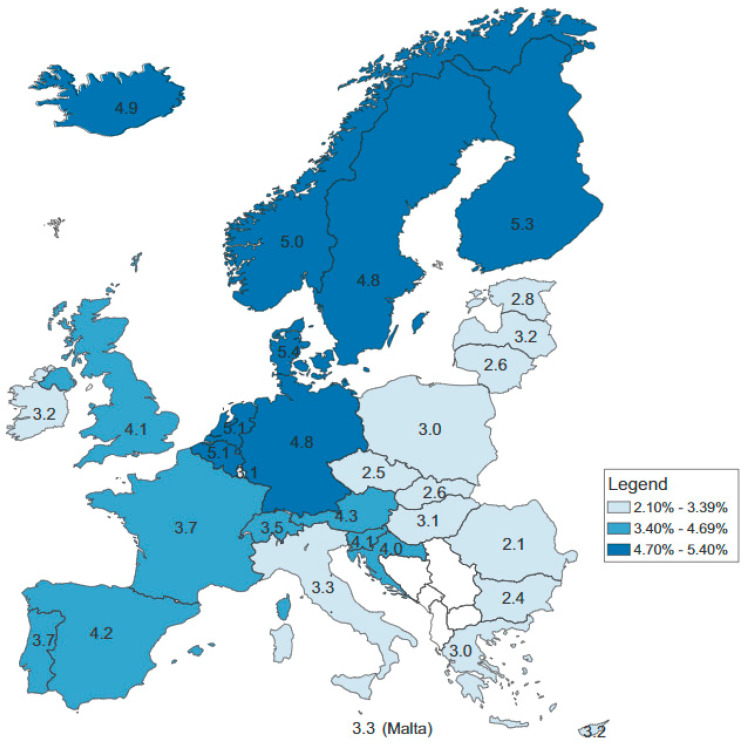
Estimated direct and indirect costs related to mental health problems across EU countries (as a % of GDP). Source: OECD estimates [7].

Employing OECD estimates, Figure 1 shows that the estimated direct and indirect costs of mental health disorders vary among countries, from around 2% of the GDP in Romania, Bulgaria and the Czech Republic to over 5% in Denmark, Finland, the Netherlands and Belgium [7]. If the mental health issues that employees deal with are not promptly treated, it is possible that business productivity suffers as a result. The efforts that governments make to improve economic conditions and the health of the population may be jeopardized as a result [8].

In order to have a well-designed strategy to tackle the urgent problem of mental health, the determinants that heavily influence it must be recognized. Therefore, the aim of the article is to evaluate the importance of mental health determinants—lifestyle, demographic factors and socio-economic status (SES)—among employees in European countries. Our research study is based on data from the second wave of the European Health Interview Survey (EHIS) for 2013–2015, which covered a sample of N = 140,791 respondents from 30 European countries. Our study advances the literature related to mental health issues in several ways. Firstly, it provides a cross-country analysis, an examination of similarities and differences among data gathered from 30 European countries. To our knowledge, there are few studies based of EHIS data that take a cross-country approach. There is a number of publications based on EHIS data related to different health issues for single European countries, such as France [9], Slovakia [10], Germany [11], Czechia [12] and Poland [13]; however, none of these is particularly devoted to the determinants of mental health. Additionally, to our knowledge, there is a very limited number of publications that use EHIS data from more than one country [14,15]. Moreover, although quite a few studies have recently been published covering a similar topic and using other cross-country datasets, such as the European Social Survey [16,17] and Life-in-Transition Survey [18,19], we found only a very small number of these on mental health that simultaneously use a multi-country approach and are based on EHIS data [20]. Secondly, we propose to view mental health issues from a rather holistic perspective, as a combination of determinants such as lifestyle, demographic factors and socio-economic status, rather than from a physiological perspective alone, which is the most common approach in the literature on mental health. Further, the analysis employs advanced, novel machine learning techniques based on gradient-boosted trees (GBTs) and SHAPley additive explanation (SHAP) values, which guarantees the validity of results in the prediction of mental health disorders.

The remainder of the paper is structured as follows: Section 2 provides a synopsis of the relevant literature related to mental health determinants. Section 3 presents methodology and data, while Section 4 summarizes the main findings. Section 5 is a discussion of the findings expanded with the contribution and limitations of the study. Finally, Section 6 presents the final conclusions.

## 2. A Review of Previous Studies on Mental Health Determinants, with a Focus on Socio-Economic Drivers

Mental health is influenced by personal attributes as well as by social, economic, cultural, educational, environmental, political and other factors; however, socio-economic determinants are not commonly discussed in the literature. The environment in which individuals are born, live, work and age, as well as the health systems to which they have access, are all socio-economic determinants that are affected by a larger number of forces: politics, economics, and social and environmental policies. Various social, economic and physical circumstances functioning at different phases of life impact a person’s mental health and several prevalent mental diseases. Socio-economic determinant frameworks focus on understanding how the circumstances in which people live and work shape their health outcomes [21]. Social determinant frameworks build upon the concept of the “social gradient”—whereby individuals with lower social status have greater health risks than those with higher status and whereby the impact of social position can accumulate over time [22]. Considering mental health, the social gradient impacts both risks of disorder and access to services and, consequently, to improved outcomes [23]. According to a review of the literature, among the socio-economic determinants that have a negative influence on mental health are: lower income [24,25,26,27] and considerable financial strain [28,29]. A two-way causality between income and health measures is identified in the fact that healthy people tend to be more productive and more likely to invest in their human capital, thereby generating higher income. On the other hand, higher income leads individuals to access health-related resources and services, with positive implications for their mental health status. 

A further socio-economic determinant is the education level, with higher levels of education being a protective factor [30]. Additionally, schools provide a secure environment for personal growth and development, the results of which can have an impact on both short- and long-term mental health [31]. A further socio-economic factor is the occupational status, with people not at work and not studying reporting greater vulnerability [32,33]. Moreover, unemployment, precarious employment and poor employment conditions continue to be routinely linked to increased psychological distress [25,26], with a higher impact on men’s mental health than on women’s [34]. The interrelated factor is related to hostile interactions with employers (i.e., injury disputes, threats of deportation) [35]. 

Another group of socio-economic determinants includes neighbourhood deprivation, revealed in, e.g., high concentrations of poverty, the perception of severe problems in the community, living in an area with high unemployment, perceived traffic stress, poor-quality built environment, violent neighbourhood crime and poorer perceptions of neighbourhood safety [36,37,38,39]. Not only poor-quality housing conditions (such as inadequate heating, overcrowding) can have negative effects on the psychological health of youths and adults [40,41] but also discrimination, whether related to race/ethnicity, immigrant status, sexual orientation and/or occupational status [42,43,44,45]. An important protective factor against common mental health disorders is a high level of social capital, defined as the resources available to individuals and to society through social relationships [46]. A high level of social capital positively affects mental health through its different components [25,27,28,47,48]:

- The structural component, i.e., the “roles, rules, precedents and procedures as well as a wide variety of networks that contribute to cooperation” [49]. In relation to mental health, structural social capital at the macro level is influenced by institutional factors, implementation mechanisms and overall health policies. At the meso level, the efficiency and effectiveness of various networks in service delivery and dissemination strategies for health-related information are crucial drivers;
- The cognitive component is made up of “mental processes and resulting ideas, reinforced by culture and ideology, particularly norms, values, attitudes, and beliefs that contribute to cooperative behaviour” [49]. Cognitive social capital, mostly socialised at the micro level, has an influence on behavioural norms in regard to mental health, including the management of risky conduct, mutual support and assistance, and unofficial channels of information transmission. It translates into trust in others, perceived emotional support and family/friend network size, sense of community belonging and shared values.

Another social factor, which may strongly impact mental health in a both positive and negative way, is familial relationships. Their role may be twofold: positive, e.g., living with family, satisfaction with family relationships and family connectedness are associated with fewer depressive symptoms [25,50]; or negative, e.g. a history of abuse and neglect from a family member is associated with symptoms of post-traumatic stress disorder (PTSD), anxiety and aggression [51,52]. Among mental health determinants related to demographic factors, the literature lists the following: age, gender, ethnicity, marital status [53], number of children in household [54] and other factors, such as perfectionism, religious orientation, self-efficacy and suicide tendencies [55].

Another group of mental health determinants is related to lifestyle. The first example is food insecurity and poor diet quality, which were demonstrated to be linked to poorer mental health in the USA and Canada [56,57,58]. Food insecurity is a likely source of long-term stress for parents trying to meet their children’s basic needs. It was also linked to mental health problems such as depression in parents. Therefore, not having enough food may affect a child’s mental health in ways related to the quality of parenting. Another commonly indicated lifestyle factor is obesity [59,60], usually measured with the BMI, which negatively affects mental health. A protective factor is physical activity, which is associated with better cognitive health and performance [60,61,62]. The relation between drinking and mental health has also been under investigation, with many studies reporting a J- or U-type association, indicating a stronger effect of moderate drinking on mental health problems in comparison with heavier drinking or abstinence [63]. On the other hand, a negative attitude towards nutrition-related activities, such as shopping and cooking frequently, increase the risk of low mental well-being [62]. Another lifestyle determinant is smoking (measured, for instance, with the number of cigarettes smoked per day or smoking status: never smoked, ex-smoker, smoker), with smoking prevalence being significantly higher among people with mental health problems than among the general population [59,60,62]. Similarly, drug use plays a negative role in mental health [62].

Additionally, the level of mental health is determined by other physical health problems, namely, the number of self-reported chronic diseases under treatment, such as heart disease, diabetes, stroke, lung disease, osteoarthritis, cancer, ulcers, intestinal problems, liver disease, epilepsy and thyroid gland disease [59].

## 3. Data and Methods

This section presents the data and methods used in this research study. After introducing the dataset, the descriptive statistics and employed methods are presented to facilitate interpretation.

### 3.1. Dataset

The second wave of the EHIS is the primary source of data for this research study. The respondents in this survey are individuals aged 15 or above living in private households in the territory of the countries. The second wave of the EHIS was conducted between 2013 and 2015 in all European Union (EU) countries together with the United Kingdom, Norway and Iceland. This newly created region is called the EU+ region. The EHIS covers the following topics: health status, access to healthcare, health determinants, socio-economic status and demography [64]. Table 1 presents variable names with definitions.

The response variable is mental health disorder, defined as at least one of the following mental health issues:

a. “sleepingproblems” is defined as the extent of having trouble falling or staying asleep or sleeping too much over the previous two weeks;
b. “failure” is defined as the extent of feeling bad about oneself or experiencing the feeling of being a failure over the previous two weeks;
c. “depressed” is defined as the extent of feeling down, depressed or hopeless over the previous two weeks.

In addition, the comprehensive variable dictionary is presented in Supplementary Materials—Supplementary A. 

### 3.2. Descriptive statistics

The original variables were reduced to 7 numerical variables and 19 categorical or binary variables (including mental health problems as a binary variable). In the data cleaning process, outliers were removed, and the dataset was tuned via data integration so as to use it as input for model estimation. After preprocessing, the below descriptive statistics were calculated for 140,791 observations. 

Table 2 presents the descriptive statistics for continuous variables. The average numbers of vegetable and fruit portions per day were 1.93 and 2.13, respectively. On average, sports activity time was around 156 min weekly; the BMI was around 25.62, and smoking amounted to 3.1 cigarettes daily. The mean number of people in the household was close to 3. Moreover, the average number of kids aged under 15 was 0.57. Tables S1–S17 in Supplementary B present the descriptive statistics for categorical variables. 

Table 3 presents the frequency of mental health problems among different EU+ countries. The highest percentages of people who declared depression or failure or sleeping problems were in the Czech Republic (37.98%), the United Kingdom (37.71%) and Portugal (36.97%). Conversely, the lowest percentages of responders with mental health disorders were in Hungary (32.47%), Malta (32.66%) and Iceland (32.97%). At the EU+ region level, 35.3% declared any type of mental health problem defined in Section 3.1, Dataset. 

### 3.3. Methods

In this research study, GBT and SHAP values were used to verify the research questions explained in the Section 1. Extreme gradient boosting (XGBoost) has been widely applied in various aspects, such as credit scoring, transportation or even medicine (for instance, for cancer morbidity prediction). Several recent studies show that GBT is the best performing algorithm in the prediction of various aspects, such as the prediction of suicide, psychological health, stress, mental health problems or depression [68,69,70,71,72,73,74,75,76,77,78,79,80,81]. Sanderson et al. [68] claim that “the gradient boosted trees model class appeared to be the most promising model class for future research”. Moreover, Cho et al. [72] suggest that “if labels are classes, the ML task is a classification problem. On the other hand, if labels are numbers in a continuous space, the task is a regression problem”. The majority of EHIS data are provided in classes, which is why we decided to use machine learning (ML) techniques to predict mental health problems. All in all, XGboost was selected due to its ability to handle missing values, to detect non-linear relationships (especially relationships in the shape of J or U-curve) and interactions between variables and to be robust to correlated features, as well as due to its interpretability thanks to SHAP values [82]. 

The SHAP value method is a feature-attribution method that assigns each feature a particular prediction value, which is useful for interpreting the prediction result. To facilitate a detailed explanation of why this research study is helpful, the combination of XGBoost modelling and SHAP values was selected as the most optimal solution. The analysis was conducted using RStudio and the xgboost package [83].

#### 3.3.1. Extreme Gradient Boosting (XGBoost) 

In 2016, Chen and Guestrinis [84] developed XGBoost based on the original framework of gradient boosting provided by Friedman in 2001 [85]. XGBoost was selected due to its high accuracy and low risk of overfitting in the application of a boosting algorithm. The simple algorithm of GBTs uses *K* additive trees to approximate the output as presented in the equation:

(1) $$y_{i}=\overset{K}{\underset{k=1}{\sum }}f_{k}\left(x_{i}\right),f_{k}\in F\;$$

where $f_{k}$ is an independent Classification and Regression Tree (CART) at *k* steps that maps input variables $x_{i}\;$to $y_{i}$ and *F* is the set of all possible CARTs. Even though a simple GBT method has the advantage of high accuracy, it is prone to overfitting. Equation (2) shows that the output is updated by adding a score of one new tree at a time:

(2) $${\hat{y}}_{i}{}^{\left(t\right)}=\overset{t}{\underset{k=1}{\sum }}f_{k}\left(x_{i}\right)={\hat{y}}_{i}^{\left(t-1\right)}+f_{k}\left(x_{i}\right)$$

Unlike GBT, in order to overcome overfitting, regularisation was added to the loss function in the objective function *Z*. Thus, XGBoost minimises regularised objective function Z defined in the equation:

(3) $$Z\left(\theta \right)=L\left(\theta \right)+\Omega \left(\theta \right)=\overset{n}{\underset{i=1}{\sum }}l\left(y_{i},{\hat{y}}_{i}\right)+\overset{K}{\underset{k=1}{\sum }}\Omega \left(f_{k}\right)$$

Finally, after taking into account Equation (2), the *t*-th iterate of objective function Z is given by the equation:

(4) $$Z^{\left(t\right)}=\overset{n}{\underset{i=1}{\sum }}l\left(y_{i},{\hat{y}}_{i}^{\left(t-1\right)}+f_{k}\left(x_{i}\right)\right)+\Omega \left(f_{k}\right)+constant$$

where:

(5) $$\Omega \left(f\right)=\gamma T+\frac{1}{2}\lambda \overset{T}{\underset{j=1}{\sum }}w_{j}^{2}$$

Regularised objective function Z contains two parts: training loss function l and regularisation term Ω. Training loss l measures the difference between predicted value ${\hat{y}}_{i}\;$and true value $y_{i}$. Regularization term Ω measures the model’s complexity, which helps to smooth the final learned weight to avoid overfitting. Equation 5 indicates that Ω contains γ—the complexity parameter of each leaf; T—the number of leaves; λ—the parameter to scale the penalty; and w—the vector of scores on leaves.

Unlike the GBT method, XGboost takes the second-order Taylor expansion in the loss function. By using the mean squared error (MSE) as a loss function, the objective function of the *t-*th tree can be derived by removing the constants. 

Unlike GBTs, XGboost provides two important techniques called shrinkage and column subsampling. The shrinkage technique scales the newly added weights at each step of boosting, thus reducing the influence of each tree and the risk of overfitting. Column subsampling chooses only a random subset of input features in building a given tree for speeding up the training process [86].

#### 3.3.2. Model Parameters

Parameter tuning in XGBoost reduces the risk of overfitting and prevents the model from becoming too complex. In practice, overfitting happens when the model begins to learn noises and random fluctuations, which leads to considering such noise as an important contributor to prediction. As might be expected, XGBoost provides several parameters that can be selected to maximize performance and protect from overfitting. In this research study, four parameters of XGBoost were tuned. A detailed description of the parameters is provided in Table 4. 

From Table 4, a grid of all possible parameters was created, and thousands of models were estimated and evaluated based on the evaluation methods discussed in Section 3.3.3.

#### 3.3.3. Model Evaluation

In this study, the following commonly used evaluation metrics were selected to measure the performance of the GBT models: Precision, Recall, F-score and Accuracy (ACC). After classification on a test set, samples were divided into four parts: True Positive (TP), False Positive (FP), True Negative (TN) and False Negative (FN). These four parts are presented as the confusion matrix in Table 5.

(6) $$\mathrm{Precision}=\frac{\mathrm{TP}}{\mathrm{TP}+\mathrm{FP}}$$

(7) $$\mathrm{Recall}\;=\;\frac{\mathrm{TP}}{\mathrm{TP}+\mathrm{FN}}$$

(8) $$F\text{-}\mathrm{score}\;=\;\frac{\mathrm{Recall}\;*\;\mathrm{Precision}\;*\;2}{\mathrm{Recall}\;+\;\mathrm{Precision}}$$

(9) $$\mathrm{Accuracy}\;=\;\frac{\mathrm{TP}\;+\;\mathrm{TN}}{\mathrm{TP}\;+\;\mathrm{FP}\;+\;\mathrm{TN}\;+\;\mathrm{FN}}$$

Generally, Hossin and Sulaiman show evaluation focus on different evaluation metrics [87]. ACC measures the ratio of correct predictions over all predictions. Next, Precision measures the positive predictions from the total prediction in a positive class, while Recall measures the fraction of positive patterns in relation to all those classified positive.

#### 3.3.4. Model Interpretation

To understand the determinants of mental health problem prediction and compare the importance of explanatory variables, SHAP values were calculated. Although XGBoost supports reporting the importance of features such as gain or split counts, these values have one disadvantage—a high value in one tree tends to show a low value in another tree. Moreover, the simple ranking of feature importance found by the XGBoost model is not enough to explain an individual prediction. Having established a clear need for a better interpretation of machine learning results, Lundberg et al. (2018) proposed the SHAP explanation method based on game theory and conditional expectation [88]. Calculating SHAP values is the only solution that satisfies the properties of Efficiency, Symmetry, Dummy and Additivity [88]. Therefore, the tree SHAP algorithm proposed by Lundberg et al. (2018) was used to explain the feature contribution to the individual mental health disorder prediction. 

## 4. Results 

This section presents results for the EU+ total population, which includes more than 140,000 employees. Table 6 presents the optimal parameters selected for final modelling for the EU+ total population after cross-validation based on the highest F-score and Precision values. Other parameters, such as booster (gbtree), objective (binary:logistic), seed (123), evaluation matrix (error), number of rounds for boosting (300) and scale_pos_weight hyperparameter (1.83), were fixed. 

**Figure 2:**
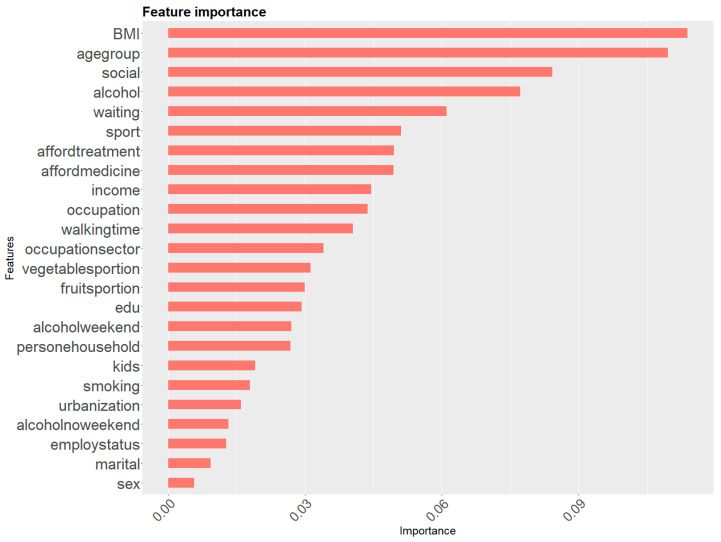
Feature importance of XGBoost model for mental health for the EU+ total population. Source: Authors’ elaboration based on microdata from EHIS 2015 [64].

Figure 2 shows feature importance in the prediction of mental health problems for EU+ total population. The ranking of the features was based on the highest gain for each feature from XGBoost after optimisation within the EU+ total population. The most important features that determined mental health disorders in the EU+ total population were BMI, age group and the number of close people to count on in case of serious personal problems. The next features with high scores were alcohol consumption, an unmet need for health care in the past 12 months due to long waiting list(s) and sports activity. An ability to afford medicine or treatment, income and occupation were also important determinants of mental health. Furthermore, the top 15 features’ ranking was rounded off by walking time, occupational sector, fruit and vegetable consumption, and education.

**Figure 3:**
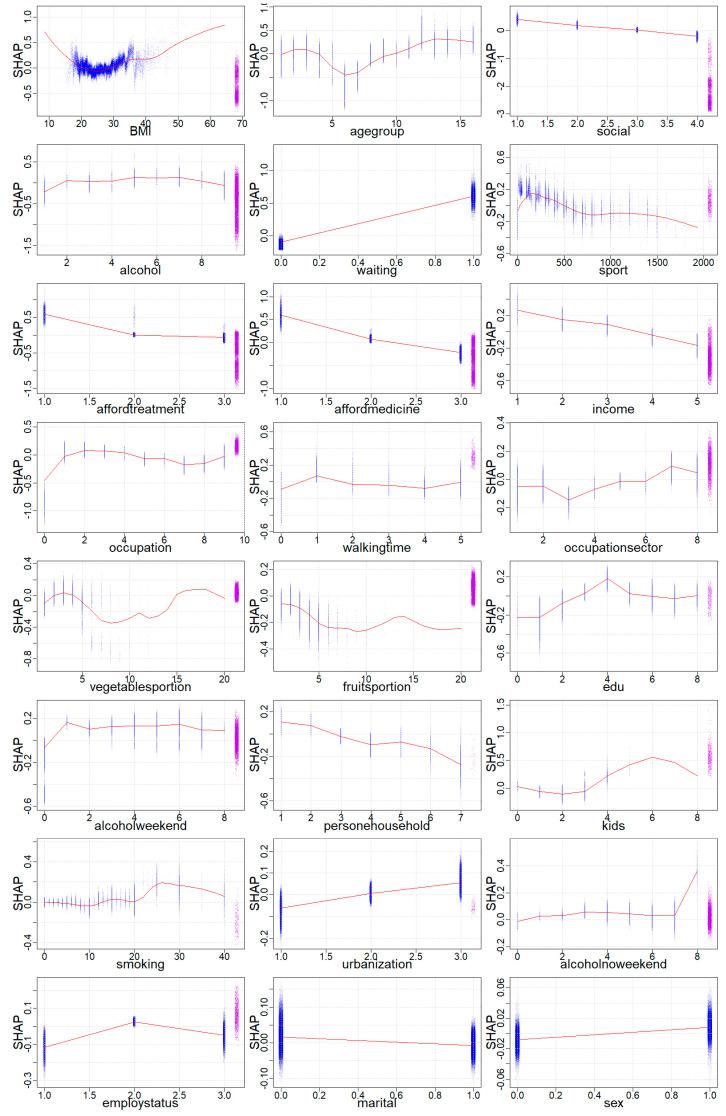
Mental health SHAP value dependency analysis for the EU+ total population. The order of variables is based on feature importance ranking from Figure 2. Source: Authors’ elaboration based on microdata from EHIS 2015 [64].

Figure 3 presents the influence of the different values of demographic, lifestyle, SES and environmental features on mental health. A normal BMI, defined in the range from 18.5 kg/m^2^ to 25 kg/m^2^, decreased the probability of mental health disorders. Figure 3 clearly shows that being underweight (BMI < 18.5 kg/m^2^) and obese (BMI > 30 kg/m^2^) increased the chance of mental health problems. The second lifestyle determinant was alcohol consumption. Although there were no significant differences in SHAP values, the lowest chance of mental health disorders was among people who had never drunk in their life (category 9) or who drank every day (category 1). Next, the impact of sports activity on mental health problems was examined. Figure 3 shows that an increase in sports activity in minutes decreased the probability of mental health problems. However, the impact of walking time showed no significant trend on mental health disorders. Moreover, Figure 3 shows the impact of fruit and vegetable consumption on the probability of mental health problems. Specifically, an increase in fruit consumption or consumption of up to eight vegetable portions per day reduced the probability of mental health problems. As shown in Table 2, the average vegetable/fruits portion consumption was around two portions per day, which confirmed that eating more fruit and vegetables reduced mental health problems. The last lifestyle determinant discussed was smoking. Figure 3 provides evidence that smoking more than 20 cigarettes per day increased the probability of mental health disorders.

The second group of determinants of mental health disorders consisted of demographic factors. Firstly, the results showed that the highest probability of mental health disorders was for employees aged 55+ (category 10 and higher). According to Figure 3, the lowest chance of mental health problems was among workers aged between 30 and 49 years old (category 5–8). In addition, workers who were married exhibited the lowest chance of mental health issues (category 1). It can be inferred from Figure 3 that the SHAP value for mental health disorders declined with the increase in the total number of persons in a household. However, a similar trend was not observed with an increase in the number of kids. Interestingly, a number of kids more or equal to four increased the chance of mental health problems. Figure 3 shows a higher probability of mental health disorders among male employees (category 1). 

Another group of mental health problem determinants consisted of SES indicators. The results presented in Figure 3 revealed that the lowest probability of mental health problems was among people who declared six or more close people to count on in case of serious personal problems (category 4). Conversely, the highest chance of mental health problems was among people who could not count on anyone in case of serious personal problems (category 1). Moreover, Figure 3 shows that the highest chance of mental health disorder was among workers who declared that they could not afford medical examination or treatment in the past 12 months (category 1) or could not afford to buy prescribed medicines (category 1). Moreover, the increase in income quintile resulted in the lowest chance of mental health disorders. Concerning occupation, the lowest chance of mental health problems was among both highly skilled blue-collar workers (category 6–7) and low-skilled blue-collar workers (category 8–9). Interestingly, Figure 3 reveals that construction workers (category 3 in the occupation sector) had the lowest chance of mental health disorders. Conversely, the lowest probability of mental health problems was among employees from public administration, defence, education, human health and social work activities (NACE codes O, P and Q—category 7) or other services (NACE codes R, S, T and U—category 8). Moreover, Figure 3 provides a more detailed insight into how education impacted mental health, showing that individuals with post-secondary but non-tertiary education (category 4) had the highest chance of mental health problems. Interestingly, the lowest chance of mental health issues was among employees with pre-primary and primary education (category 0–1). Concerning employment status, Figure 3 reveals that the highest chance for mental health disorder was among employees with a permanent work contract (category 2). 

The impact of the environmental factors on mental health disorders was examined next. Figure 3 suggests that an unmet need for health care in the past 12 months due to long waiting lists (category 1) substantially increased the chances of mental health problems. Interestingly, the highest chance of mental health disorders was among workers from areas of low population density (category 3). 

Table 7 presents the model evaluation metrics presented in Section 3.3.3. Based on Accuracy criteria, observations for 65% of employees were correctly predicted in terms of absence or presence of mental health disorders among total observations. Recall is the ratio of correctly predicted workers with mental health problems to all actual employees with mental health disorders. This model achieved a Recall of 0.6439, which is good for this analysis and, importantly, is above 0.5 (random choice).

## 5. Discussion

While psychological determinants are widely discussed in the context of mental health disorders, there is still little investigation from the multi-dimensional perspective to comprehensively understand the impact of lifestyle and demographic factors combined with socio-economic status on mental health disorders among employees in EU countries. The ambition of the article follows the third goal of sustainable development, which is to ensure healthy lives and promote well-being for all at all ages [89]. This article may be considered as a significant contribution to the growing empirical evidence on the subject matter by exploring the impact of various factors on mental health disorders defined as subjective sleeping problems or a feeling of failure or depression. 

Data from the second wave of the EHIS for 2013–2015, which covered a sample of more than 140,000 workers from 30 countries in the EU + region (the EU together with the United Kingdom, Norway and Iceland) were employed in this research study. The data analysis was carried out with the use of novel machine learning techniques, particularly, GBTs and SHAP values. Recent studies showed that the GBT algorithm is the best performing algorithm in the prediction of various mental health problems [68,69,70,71,72,73,74,75,76,77,78,79,80,81].

The key results of various factors on mental health disorders revealed that the most important features determining mental health problems for the EU+ population of the surveyed employees were the BMI, the age group and the number of close people to count on in case of serious personal problems. Figure 2 shows that alcohol consumption, an unmet need for health care in the past 12 months due to long waiting lists and sports activity played a vital role in the prediction of mental health issues. Importantly, being able to afford medicine or treatment, income and occupation were also important determinants of mental health. However, the results of this research study indicated that lifestyle factors (BMI, alcohol, sports activity) were the most important features in predicting mental health disorders. It was proven that illness and disease were particularly pronounced among low-SES groups and that a healthy lifestyle had significant positive effects on both physical and psychological health [90]. The second important group of mental disorders determinants consisted of socio-economic features (social support, income, occupation, being able to afford medicine or treatment). The third important group of determinants to determine mental health disorders consisted of demographic factors (e.g., age group). Importantly, this study revealed that socio-economic determinants ranked second for importance in predicting mental health disorders, while lifestyle played a crucial role. The results confirmed the model of the cycle of mental well-being provided by the WHO for Europe [91]. European Mental Health Action Plan 2013–2020 suggests that lifestyle factors such as poor diet, smoking and alcohol consumption increase the incidence of mental health disorders. Moreover, modelling the association between lifestyle and mental health outcomes is more complex at an individual level rather than at a population level [91]. The WHO confirms that socio-economic status (e.g., wealth), demographics (e.g., age) and health system characteristics (e.g., access and affordability) impact mental health well-being [91]. 

Firstly, the relationship between lifestyle and mental health was investigated. Figure 3 shows that being underweight (BMI < 18.5 kg/m^2^) and obese (BM > 30 kg/m^2^) increased the chance of mental health problems, which is consistent with the literature [59,60]. Moreover, there were no significant differences in SHAP values, as the lowest chance of mental health disorders was among people who had never drunk alcohol (category 9) or who drank it every day (category 1). However, Aceijas et al. [62] suggested that the number of alcoholic drinks consumed per day plays a negative role in mental health. Generally, alcohol consumption is associated with one or more diagnosed mental health conditions [92], which was not visible in this research study. El-Guebaly [93], who provided a robust review of studies around the association between moderate drinking and mental health, indicated that most studies report a “J-shaped curve,” with positive self-reports of subjective mental health associated with moderate drinking but not with heavier drinking. Importantly, the association between alcohol and mental health should be measured in the short and long term. Some people may drink alcohol to try to relieve the symptoms of mental disorders. In the short term, heavy drinking interferes with chemicals in the brain that are vital for good mental health. However, in the long run, the effect of alcohol may lead to anxiety and depression [94]. Moreover, the literature review found that physical activity is a protective factor against mental disorders [60,61,62]. The results of this study, presented in Figure 3, noted that a weekly increase in sports activity in minutes reduces the probability of mental health problems. The impact of diet was also examined. Figure 3 shows that an increase in fruit consumption or consumption of up to eight vegetable portions per day reduced the probability of mental health problems. As shown in Table 2, the average vegetable/fruit portion consumption was around two portions per day, which confirmed that eating more fruit and vegetables reduced mental health problems. These results confirmed the outcomes from another study in Australia, which found that an increase in fruit and vegetable consumption to eight portions a day was predictive of increased happiness, life satisfaction and well-being [95]. In the case of the USA and Canada, poor diet quality was linked to poorer mental health [56,57,58]. The last investigated lifestyle determinant was smoking. Figure 3 provides evidence that smoking more than 20 cigarettes daily increased the probability of mental health disorders. Explanations of the above result can be found in the literature. It proved that the prevalence of smoking was significantly higher among people with mental health problems than among the general population [59,60,62]. In terms of lifestyle determinants, the crucial findings from this study were that the impact of alcohol consumption and smoking on mental health was ambiguous. There is an ongoing debate about the relationship between the two [96]. While consuming alcohol or smoking, someone may feel relaxed, less anxious and more confident [94], which is why mental problems can lead to a greater tendency to consume alcohol or smoke. Further research may require stratification by gender [97]. 

Secondly, the association between socio-economic status and mental health was examined. Importantly, Figure 3 reveals that the lowest probability of mental health problems was among people who declared six or more close people to count on in case of serious personal problems (category 4). It was consistent with the literature studies that found protective factors against common mental health disorders in high levels of social capital, defined as the resources available to individuals and to society through social relationships [25,27,28,46,47,48,50]. The results presented in Figure 3 revealed that the highest probability of mental health disorders was among workers who declared that they could not afford medical examination or treatment in the past 12 months (category) or could not afford to buy prescribed medicines (category 1). In addition, lower income (first quintile) workers had the highest chance of mental health disorders. This is confirmed by several studies that found that mental illness was often associated with lower income [24,25,26,27] and considerable financial strain [28,29]. Concerning occupation, the lowest chance of mental health problems was among both high-skilled blue-collar workers (category 6–7) and low-skilled blue-collar workers (category 8–9). One of the most recent studies by Väisänen et al. [98] suggested that blue-collar workers had significantly higher health risks than white-collar workers, although this study did not only focus on mental health problems. In addition, Schreuder et al. [99] reported similar health complaints among white- and blue-collar workers, which is why further research analysing white- and blue-collar workers together with their socio-economic position instead of working conditions would be recommended [99]. In addition, this research study examined the impact of education on mental health. Figure 3 shows that employees with post-secondary but non-tertiary education (category 4) had the highest chance of mental health problems. Interestingly, the lowest chance of mental health issues occurred among employees with pre-primary and primary education (categories 0–1). However, Allen et al. [30] found that higher levels of education were a protective factor against mental health disorders. Conversely, a recent study showed that people with higher education had more psychological distress than persons with low and medium education; however, other SES features, such as age, employment status and social support, should be taken into account [100]. Moreover, several studies suggested that low education was associated with fewer psychosocial resources, which in combination with daily stress work as pathways between education and depressive symptoms [101,102,103]. Importantly, few studies showed no significant impacts of education on mental health [103,104,105]. Further research focused on longitudinal studies is crucial to identify causality. The results of this research study might be explained by mental health literacy, which is commonly defined as “knowledge and beliefs about mental disorders, which aid their recognition, management or prevention” [106]. Due to self-reported data, the inadequate cognition of mental health problems might be related to low levels of education [107,108,109]. As regards employment status, Figure 3 shows that there was a higher chance of mental health disorders among employees with a permanent job. The most important findings suggested that unemployment or temporary employment were linked to increased psychological distress [25,26]. However, there is conflicting evidence on an impact of a permanent work contract on self-rated health status or mental health (e.g., depression), with some researchers revealing negative influence [110,111], but with most of them showing a significantly higher risk of developing depression or other mental health problems among unemployed or precariously employed [112,113]. Moreover, the latest meta-analysis of longitudinal studies revealed that evidence of the impact of temporary employment on mental health is of low quality [114]. The findings from the European Social Survey from 2018 suggested that sociodemographic variables had a significant impact on happiness and life satisfaction in European countries [17]. Importantly, an individual’s social relationships and an increase in household income were the variables that contribute the most to the happiness scale [17]. The strength of social relationships and the ability to live on a household income were the most powerful predictors of life satisfaction. As a result of this research study, employers should take into account the socio-economic status of their workers, looking in particular at social support and the ability to afford medicine or treatment in order to prevent mental health problems. This is reinforced by the fact that inequity in mental health service usage is driven by the education level [91,115]. 

The contemporary nature of mental health disorders requires a multi-dimensional perspective, which is why demographic factors and environmental aspects were examined. Our results showed that age was the second most important factor (after BMI) that determined the mental health disorders among European employees. Figure 3 shows that employees aged 55+ (category 10 and higher) had a higher probability of mental health issues. Several studies showed that 15–20% of people aged 55+ suffer from mental disorders [53,116,117], which confirms our results. Moreover, the lowest chance of mental health issues was among workers who were married (category 1). Figure 3 shows that the SHAP value for mental health disorders declined with the growth in the total number of persons in a household. However, this study revealed a higher probability of mental health disorders among male employees (category 1). According to a study of more than 43,000 people, women were more likely to be diagnosed with anxiety or depression, while men tended toward substance abuse or antisocial disorders [118]. One of the latest systematic reviews underlined the important role of sex in the development of the prevention of mental disorders [119]. Figure 3 shows that there was a higher chance of mental health disorders for workers from less densely populated areas (category 3). Importantly, access to medical services in such areas is limited. However, Gruebner et el. [120] suggested that the risk of serious mental illness is generally higher in cities than in rural areas. Moreover, Figure 3 reveals that an unmet need for health care in the past 12 months due to long waiting lists (category 1) substantially increased the chances of mental health problems. 

This study revealed that lifestyle (e.g., BMI, alcohol, physical activity), socio-economic status (e.g., social contact and income) and demographics (e.g., age) may constitute critical determinants of mental health disorders among employees. A systematic review provided by Lund et al. [121] suggested a strong association between age or social support and mental disorders, especially depression. In addition, the result of a study carried out on a representative population survey among close to 8000 representatives of the German population showed that the body mass index within the range of normal to overweight resulted in better mental health [122]. Therefore, continuing to include multi-dimensional measures in further research on risk factors may shed new light on the collective impact of these variables.

To conclude, the recommendation for employers is to pay special attention to the lifestyle features of their employees, such as the BMI, the physical activity and the diet of their workers and, as such, promote a healthy way of life. Some preventative actions are also required (such as providing time-management coaching, instituting morning briefings, providing quiet spaces, fostering a sense of value among employees, and educating them about the signs of depression and burnout), which may mitigate the possibility of mental health disorders and result in lower chances of sickness absence due to mental illness [123]. Special programmes, including outplacement assistance, has to be offered for older employees, where the probability of mental health disorders is the highest [124].

The study makes a significant contribution to the body of knowledge on mental health determinants, as it looks at the causes of employees’ mental health problems in a multi-dimensional way and is unique, in that it was based on a large sample of N = 140,791 respondents from 30 European countries. However, the data were gathered as a snapshot from only one wave of the EHIS survey (the second wave in 2013–2015), which was a limitation of this research study. Moreover, the panel was not established, as EHIS 1 and EHIS 2 do not provide enough data points to run this type of analysis (only two data points in time). This research study was a cross-sectional study, which makes the analysing of causality impossible, so causal conclusions were not able to be made. Moreover, the literature review demonstrates different types of determinants of mental health disorders, but the conclusions are only based on the variables studied, as corresponding to EHIS data. Additionally, the EHIS survey is declarative, so its accuracy, especially around sensitive issues such as alcohol consumption or sport activity, cannot fully reflect reality. Moreover, characteristics of work including the psychosocial workplace that has been shown to be related to mental health outcomes were not included as they are not covered by EHIS data.

Furthermore, since the diagnoses of mental disorders can only be made by physicians during clinical interviews, our findings should not be interpreted as clinical diagnosis but rather as data around possible diagnoses of the factors influencing mental health. Since all data were self-reported by employees, it may restrict the applicability of our findings. Our conclusions may also not apply to youngsters, unemployed and those who are retired. We also decided not to include variables related to physical health problems. Moreover, EHIS 2 does not include variables related to working conditions (anxiety or work–life balance issues). Additionally, the study does not cover the interrelations between several mental health determinants and their possible enforced influence. The directions of the influence need to be further investigated in future longitudinal investigations. Third, since 2017, there have been significant changes in mental health patterns, particularly during and immediately following the COVID-19 pandemic. Once the data of EHIS-3 are available, it will be crucial to confirm our results. 

Despite its flaws, the study of determinants of mental health disorders was conducted on a relatively large dataset of respondents from various EU+ countries (>140,000 respondents). Importantly, finding determinants of mental health problems was based on a multi-dimensional approach including lifestyle, demographic factors and socio-economic status, which offer important guidance for upcoming policies and treatments aimed at reducing mental health difficulties among European employees. However, further research should be performed to ensure a better understanding of the direction of influence between mental health, lifestyle or SES. Further studies should also investigate changes in mental health over time in order to reveal key features that have an impact on the deterioration of mental health. Lastly, further research is needed to identify the effectiveness of policy intervention in safeguarding the mental health of employees.

## 6. Conclusions

The results of this study prove that ensuring the healthy living and well-being of employees is only possible through a comprehensive overview of all various determinants of mental health disorders. Generally, lifestyle factors play the most important role as protective factors against mental diseases. Therefore, employers should take care of the BMI and physical activity of their workers. Importantly, the results show that demographic factors such as the age cohort are crucial to improving the effectiveness of the implementation of certain policy actions. All in all, the socio-economic status was the second most important determinant of mental health disorders due to social contacts. Moreover, the impact of income and occupation together with other attributes may play a vital role in the complex understanding of mental health problems. More in-depth knowledge about different determinants of mental health diseases across diverse socio-economic groups might guide policymakers to the effective development of health-promoting programmes. Interventions should be designed together with experts from governmental and non-governmental organizations and take into account a complete model of lifestyle, demographics, and environmental and socio-economic factors at an individual level to improve the mental health of society as a whole. Effective policy actions are especially needed in mitigating the severe impact of the COVID-19 pandemic on mental health, manifested as a decrease in mental resilience on an unprecedented scale. The need for collecting good-quality data should also be emphasised as a measure that may allow the preparation of adequate mental health response plans at the national and international levels. 

## Figures and Tables

**Table 1:** Explanatory variables employed in further research.

Type of Variable	Variable Name	Variable Definition
Demographic factors	sex	female (0) or male (1)
	agegroup	16 different cohorts starting from 15 years old up to the 85+ group
	marital	marital status (married or not married, widowed, divorced)
	kids	number of persons in the household aged from 0 to 15
	personhousehold	total number of persons in the household
Lifestyle	smoking	average number of cigarettes smoked per day
	alcohol	frequency of consumption of an alcoholic drink of any kind (beer, wine, cider, spirits, cocktails, premixes, liqueurs, homemade alcohol, etc.) in the past 12 months
	alcoholweekend	number of alcoholic (standard) drinks consumed on average on one of the days (Friday to Sunday)
	alcoholnoweekend	number of alcoholic (standard) drinks consumed on average on one of the days (Monday to Thursday)
	sport	time spent doing sports, fitness or recreational (leisure) physical activities in a typical week (in minutes)
	walkingtime	time spent walking to get to and from places on a typical day
	fruitsportion	the number of portions of fruit a day, excluding juice
	vegetablesportion	number of portions of vegetables or salad a day, excluding juice and potatoes
	BMI	body mass index—weight/height^2^ expressed in units of kg\m^2^
Socio-economic status (SES)	education	education level based on ISCED-2011 classification from early childhood development to doctoral level [65]
	occupation	International Standard Classification of Occupations (ISCO-08) classification [66]
	occupationsector	economic sector in employment based on NACE Rev. 2 [67]
	employstatus	employment status
	income	net monthly equivalised income of the household in quantiles
	social	number of close people to count on in case of serious personal problems
	affordtreatment	could not afford medical examination or treatment in the past 12 months
	affordmedicine	could not afford prescribed medicines in the past 12 months
Environmental factors	waiting	unmet need for healthcare in the past 12 months due to long waiting list(s)
	distance	unmet need for healthcare in the past 12 months due to distance or transportation problems
	urbanisation	degree of urbanisation

Source: Authors’ elaboration based on microdata from EHIS 2015 [64].

**Table 2:** Descriptive statistics for continuous variables.

Variable	Mean	St.Dev ^1^	Min	Max
vegetablesportion	1.93	1.25	1.00	20.00
fruitsportion	2.13	1.21	1.00	20.00
smoking	3.10	6.81	0.00	40.00
sport	156.25	259.50	0.00	1930.00
kids	0.57	0.87	0.00	8.00
personhousehold	2.93	1.32	2.00	7.00
BMI	25.62	4.4	10.12	69.20

^1^ Standard deviation. Source: Authors’ elaboration based on microdata from EHIS 2015 [64].

**Table 3:** Mental health disorders frequency in EU+ countries.

Country	Mental Health Problem ^1^	Depression	Failure	Sleeping Problems
Czech Republic	37.98%	18.20%	13.34%	30.57%
United Kingdom	37.71%	18.35%	13.53%	29.73%
Portugal	36.97%	17.81%	12.46%	29.23%
Greece	36.43%	17.27%	12.26%	29.26%
Norway	36.00%	17.58%	11.53%	28.74%
Estonia	35.99%	17.74%	12.62%	28.49%
Slovenia	35.88%	17.70%	12.06%	27.15%
Lithuania	35.86%	17.80%	11.40%	29.42%
Slovakia	35.85%	17.22%	12.76%	27.51%
Ireland	35.70%	18.21%	13.18%	27.45%
Italy	35.61%	17.20%	12.26%	27.69%
Latvia	35.55%	17.08%	12.24%	27.37%
Belgium	35.53%	17.61%	12.04%	27.78%
Finland	35.52%	17.44%	11.19%	28.49%
Denmark	35.47%	17.72%	11.86%	27.82%
Netherlands	35.41%	16.61%	12.14%	28.49%
Spain	35.36%	17.06%	12.49%	27.89%
Croatia	35.16%	17.88%	13.16%	27.82%
Sweden	35.08%	17.07%	11.46%	27.42%
France	35.07%	17.04%	11.94%	26.65%
Germany	35.00%	17.54%	11.68%	26.99%
Poland	34.88%	17.32%	11.23%	26.48%
Bulgaria	34.04%	16.77%	11.49%	26.03%
Cyprus	33.96%	17.18%	11.28%	25.73%
Austria	33.44%	15.73%	10.02%	25.44%
Luxembourg	33.30%	16.08%	11.72%	26.27%
Romania	33.25%	16.15%	10.51%	25.31%
Iceland	32.97%	15.39%	11.50%	25.41%
Malta	32.66%	15.96%	9.71%	25.29%
Hungary	32.47%	15.22%	10.81%	24.88%
EU +	35.30%	17.21%	11.91%	27.47%

^1^ Mental health problem is defined as the extent of feeling, over the previous two weeks, at least one of the following: having trouble falling or staying asleep or sleeping too much; feeling bad about oneself or experiencing the feeling of being a failure; feeling down, depressed or hopeless. Source: Authors’ elaboration based on microdata from EHIS 2015 [64].

**Table 4:** Parameters selected for optimization process under XGBoost algorithm.

Hyperparameter	Description	From	To	By
Maximum depth	Maximum number of edges from the node to the tree’s root node	2	12	1
Minimum child weight	Minimum sum of weights of all observations required in a child	0	12	1
Subsample	Ratio of observations for sampling to construct each tree	0.6	0.9	0.01
Learning rate	Amount of updating weights	0.01	0.3	0.01

Source: Authors’ elaboration.

**Table 5:** Confusion matrix for binary classification.

		Predicted condition	
		Predicted Positive	Predicted Negative
Actual condition	Actual Positive	True Positive (TP)	False Negative (FN)
	Actual Negative	False Positive (FP)	True Negative (TN)

Source: Authors’ elaboration.

**Table 6:** Optimal hyperparameters for XGBoost mental health model for the EU+ total population.

Hyperparameter	Optimal Value
Maximum depth	7
Minimum child weight	8
Subsample	0.06
Learning rate	0.05

Source: Authors’ elaboration based on microdata from EHIS 2015 [64].

**Table 7:** Final model evaluation metrics.

Evaluation Metric	Value
Precision	0.4878
Recall	0.6439
F-score	0.2775
ACC	0.6444

Source: Authors’ elaboration based on microdata from EHIS 2015 [64].

## Data Availability

The study was conducted based on confidential data from Eurostat from the European Health Interview Survey (EHIS) under Research Project Proposal RPP 117/2020-EHIS.

## Supplementary Materials

The following supporting information can be downloaded at: https://www.mdpi.com/article/10.3390/ijerph191911913/s1, Supplementary A presents the variable dictionary. Supplementary B presents the descriptive statistics for categorical variables.
