# Supplementary material for: Lifestyle, Demographic and Socio-Economic Determinants of Mental Health Disorders of Employees in the European Countries
Source: Int J Environ Res Public Health. 2022 Sep 21;19(19):11913. doi: 10.3390/ijerph191911913 (PMC9565551; doi:10.3390/ijerph191911913)
Supplement: Supplementary file 1 [file ijerph-19-11913-s001.zip › ijerph-1867487-supplementary.pdf]

A Variable dictionary

This section presents the variables repository created based on EHIS dataset. The final list of variables included in the study is given below.

1. sex (binary)
   (a) 0 — female
   (b) 1 — male
2. $agegroup$ (categorical)
   (a) 1 — 15 – 17 years old
   (b) 2 — 18 – 19 years old
   (c) 3 — 20 – 24 years old
   (d) 4 — 25 – 29 years old
   (e) 5 — 30 – 34 years old
   (f) 6 — 35 – 39 years old
   (g) 7 — 40 – 44 years old
   (h) 8 — 45 – 49 years old
   (i) 9 — 50 – 54 years old
   (j) 10 — 55 – 59 years old
   (k) 11 — 60 – 64 years old
   (l) 12 — 65 – 69 years old
   (m) 13 — 70 –74 years old
   (n) 14 — 75 –79 years old
   (o) 15 — 80 – 84 years old
   (p) 16 — 85+ years old
3. $marital$ (binary)
   (a) 0 — not married, widowed, divorced
   (b) 1 — married or in a registered partnership
4. $kids$ (continuous) — Number of persons aged from 0 to 15
5. $personehousehold$ (continuous) — Total number of persons in household

6. $social$ (categorical) — number of close people to count on in case of serious personal problems
    (a) 1 — None
    (b) 2 — 1 or 2
    (c) 3 — 3 to 5
    (d) 4 — 6 or more
7. $smoking$ (continuous) — average number of cigarettes a day
8. $alcohol$ (categorical) — Frequency of consumption of an alcoholic drink of any kind (beer, wine, cider, spirits, cocktails, premixes, liqueurs, homemade alcohol. . .) in the past 12 months
    (a) 1 — Every day or almost
    (b) 2 — 5 – 6 days a week
    (c) 3 — 3 – 4 days a week
    (d) 4 — 1 – 2 days a week
    (e) 5 — 2 – 3 days in a month
    (f) 6 — Once a month
    (g) 7 — Less than once a month
    (h) 8 — Not in the past 12 months, as I no longer drink alcohol
    (i) 9 — Never, or only a few sips or tries, in my whole life
9. $alcoholweekend$ (categorical) — Number of alcoholic (standard) drinks on average on one of the days (Friday — Sunday)
    (a) 1 — 16 or more drinks
    (b) 2 — 10 – 15 drinks a day
    (c) 3 — 6 – 9 drinks a day
    (d) 4 — 4 – 5 drinks a day
    (e) 5 — 3 drinks a day
    (f) 6 — 2 drinks a day
    (g) 7 — 1 drink a day
    (h) 8 — 0 drink a day

10. *alcoholnoweekend* (categorical) — Number of alcoholic (standard) drinks on average on one of the days (Monday to Thursday)

    (a) 1 — 16 or more drinks
    (b) 2 — 10 – 15 drinks a day
    (c) 3 — 6 – 9 drinks a day
    (d) 4 — 4 – 5 drinks a day
    (e) 5 — 3 drinks a day
    (f) 6 — 2 drinks a day
    (g) 7 — 1 drink a day
    (h) 8 — 0 drink a day

11. *sport* (continuous) — Time spent on doing sports, fitness or recreational (leisure) physical activities in a typical week (minutes)
12. *walkingtime* (categorical) — Time spent on walking to get to and from places on a typical day

    (a) 1 — 10 – 29 minutes per day
    (b) 2 — 30 – 59 minutes per day
    (c) 3 — 1 hour to less than 2 hours per day
    (d) 4 — 2 hours to less than 3 hours per day
    (e) 5 — 3 hours or more per day

13. *fruitsportion* (continuous) — Number of portions of fruit a day, excluding juice
14. *vegetablesportion* (continuous) — Number of portions of vegetables or salad, excluding juice and potatoes a day
15. *BMI* (continuous) — Body mass index expressed in units of ($\mathrm{kg/m^2}$)
16. *edu* (categorical)

    (a) 0 — Early childhood development, pre-primary education
    (b) 1 — Primary education
    (c) 2 — Lower secondary education
    (d) 3 — Upper secondary education
    (e) 4 — Post-secondary but non-tertiary education
    (f) 5 — Tertiary education; short-cycle

    (g) 6 — Tertiary education; bachelor level or equivalent
    (h) 7 — Tertiary education; master level or equivalent
    (i) 8 — Tertiary education; doctoral level or equivalent

17. *occupation* (categorical)
    (a) 0 — armed forces occupations
    (b) 1 — managers
    (c) 2 — professionals
    (d) 3 — technicians and associate professionals
    (e) 4 — clerical support workers
    (f) 5 — services and sales workers
    (g) 6 — skilled agricultural, forestry and fishery workerks
    (h) 7 — craft and related trades workers
    (i) 8 — plant and machine operators and assemblers
    (j) 9 — elementary occupations

18. *occupationsector* (categorical)
    (a) 1 — Agriculture, forestry and fishing (NACE code A)
    (b) 2 — Manufacturing, mining and quarrying and other industry (NACE codes B, C, D and E)
    (c) 3 — Construction (NACE code F)
    (d) 4 — Wholesale and retail trades, transport, information and communication, accommodation and food service activities (NACE codes G, H, I and J)
    (e) 5 — Financial and insurance activities (NACE code K)
    (f) 6 — Business services and real estate activities (NACE codes L, M and N)
    (g) 7 — Public administration, defence, education, human health and social work activities (NACE codes O, P, and Q)
    (h) 8 — Other services - NACE codes R, S, T and U
    (i) 9 — Unknown sector

19. *employstatus* (categorical)
    (a) 1 — Self employed
    (b) 2 — Employee with a permanent job/work contract of unlimited duration

(c) 3 — Employee with a temporary job/work contract of limited duration

20. $income$ (categorical)
    (a) 1 — Below 1st quintile
    (b) 2 — Between 1st quintile and 2nd quintile
    (c) 3 — Between 2nd quintile and 3rd quintile
    (d) 4 — Between 3rd quintile and 4th quintile
    (e) 5 — Between 4th quintile and 5th quintile
21. $affordtreatment$ (categorical)
    (a) 1 — Could not afford medical examination or treatment in the past 12 months
    (b) 2 — Could afford medical examination or treatment in the past 12 months
    (c) 3 — No need to buy medical examination or treatment in the past 12 months
22. $affordmedicine$ (categorical)
    (a) 1 — Could not afford prescribed medicines in the past 12 months
    (b) 2 — Could afford prescribed medicines in the past 12 months
    (c) 3 — No need to buy prescribed medicines in the past 12 months
23. $waiting$ (binary) — Unmet need for healthcare in the past 12 months due to long waiting list(s)
    (a) 0 — No
    (b) 1 — Yes
24. $distance$ (binary) — Unmet need for healthcare in the past 12 months due to distance or transportation problems
    (a) 0 — No
    (b) 1 — Yes
25. $urbanization$ (categorical)
    (a) 1 — Densely-populated area Everybody unaltered
    (b) 2 — Intermediate-populated area
    (c) 3 — Thinly-populated area
26. $sleepingproblems$ (binary) — Extent of having trouble falling or staying asleep, or sleeping too much over the last 2 weeks

(a) 0— Not at all

(b) 1 — at least several days up to nearly every day

27. $failure$ (binary) — Extent of feeling bad about yourself, feeling being a failure over the last 2 weeks

    (a) 0— Not at all

    (b) 1 — at least several days up to nearly every day

28. $depressed$ (binary) — Extent of feeling down, depressed or hopeless over the last 2 weeks

    (a) 0— Not at all

    (b) 1 — at least several days up to nearly every day

B Descriptive statistics for categorical variables

This section presents the descriptive statistics for categorical variables used in this study.

Table S1: Frequency table for marital. *Source:* own calculations

Value	Frequency	Percent
1	86296.00	59.40
0	58885.00	40.60
Total	145181.00	100.00

Table S2: Frequency table for edu. *Source:* own calculations

Value	Frequency	Percent
3	60942.00	42.00
7	19964.00	13.80
2	18417.00	12.70
6	17505.00	12.10
5	11949.00	8.20
1	6812.00	4.70
4	6699.00	4.60
8	1728.00	1.20
NA	636.00	0.40
0	529.00	0.40
Total	145181.00	100.00

Table S3: Frequency table for occupation. *Source:* own calculations

Value	Frequency	Percent
2	29489.00	20.30
5	22638.00	15.60
3	21580.00	14.90
7	15968.00	11.00
4	13152.00	9.10
9	11135.00	7.70
8	10179.00	7.00
1	10122.00	7.00
6	6464.00	4.50
NA	3703.00	2.60
0	751.00	0.50
Total	145181.00	100.00

Table S4: Frequency table for occupationsector. *Source:* own calculations

Value	Frequency	Percent
7	40303.00	27.80
4	35392.00	24.40
2	23935.00	16.50
6	11968.00	8.20
3	8444.00	5.80
1	8364.00	5.80
8	8018.00	5.50
5	4719.00	3.30
NA	4038.00	2.80
Total	145181.00	100.00

Table S5: Frequency table for employstatus. *Source:* own calculations

Value	Frequency	Percent
2	107895.00	74.30
1	21054.00	14.50
3	14322.00	9.90
NA	1910.00	1.30
Total	145181.00	100.00

Table S6: Frequency table for fullparttime. *Source:* own calculations

Value	Frequency	Percent
1	121279.00	83.50
2	23902.00	16.50
Total	145181.00	100.00

Table S7: Frequency table for income. *Source:* own calculations

Value	Frequency	Percent
5	39098.00	26.90
4	34299.00	23.60
3	27147.00	18.70
2	20500.00	14.10
1	15713.00	10.80
NA	8424.00	5.80
Total	145181.00	100.00

Table S8: Frequency table for affordtreatment. *Source:* own calculations

Value	Frequency	Percent
2	94937.00	65.40
3	34149.00	23.50
NA	10803.00	7.40
1	5292.00	3.60
Total	145181.00	100.00

Table S9: Frequency table for affordmedicine. *Source:* own calculations

Value	Frequency	Percent
2	94273.00	64.90
3	36211.00	24.90
NA	10867.00	7.50
1	3830.00	2.60
Total	145181.00	100.00

Table S10: Frequency table for social. *Source:* own calculations

Value	Frequency	Percent
3	63476.00	43.70
2	38568.00	26.60
4	36349.00	25.00
NA	4481.00	3.10
1	2307.00	1.60
Total	145181.00	100.00

Table S11: Frequency table for walkingtime. *Source:* own calculations

Value	Frequency	Percent
1	52538.00	36.20
0	43027.00	29.60
2	28329.00	19.50
3	12162.00	8.40
5	4889.00	3.40
4	3615.00	2.50
NA	621.00	0.40
Total	145181.00	100.00

Table S12: Frequency table for alcohol. *Source:* own calculations

Value	Frequency	Percent
4	25468.00	17.50
NA	25393.00	17.50
5	20522.00	14.10
7	18183.00	12.50
6	15081.00	10.40
9	10703.00	7.40
3	10520.00	7.20
1	8229.00	5.70
8	7907.00	5.40
2	3175.00	2.20
Total	145181.00	100.00

Table S13: Frequency table for alcoholweekend. *Source:* own calculations

Value	Frequency	Percent
0	105958.00	73.00
NA	11433.00	7.90
6	7427.00	5.10
7	6684.00	4.60
5	5320.00	3.70
4	4505.00	3.10
3	2526.00	1.70
2	918.00	0.60
1	295.00	0.20
8	115.00	0.10
Total	145181.00	100.00

Table S14: Frequency table for alcoholnoweekend. *Source:* own calculations

Value	Frequency	Percent
0	105958.00	73.00
7	12075.00	8.30
NA	10682.00	7.40
6	7707.00	5.30
5	3849.00	2.70
4	2671.00	1.80
3	1359.00	0.90
2	434.00	0.30
8	287.00	0.20
1	159.00	0.10
Total	145181.00	100.00

Table S15: Frequency table for waiting. *Source:* own calculations

Value	Frequency	Percent
0	127842.00	88.10
1	17339.00	11.90
Total	145181.00	100.00

Table S16: Frequency table for distance. *Source:* own calculations

Value	Frequency	Percent
0	142899.00	98.40
1	2282.00	1.60
Total	145181.00	100.00

Table S17: Frequency table for urbanization. *Source:* own calculations

Value	Frequency	Percent
1	52275.00	36.00
3	48974.00	33.70
2	43757.00	30.10
NA	175.00	0.10
Total	145181.00	100.00
